# Nociception-specific blink reflex: pharmacology in healthy volunteers

**DOI:** 10.1186/s10194-015-0568-7

**Published:** 2015-10-08

**Authors:** JCA Marin, AR Gantenbein, K. Paemeleire, H. Kaube, PJ Goadsby

**Affiliations:** NIHR-Wellcome Trust King’s Clinical Research Facility, King’s College Hospital, London, UK; Department of Basic and Clinical Neuroscience, Institute of Psychiatry, Psychology and Neuroscience, King’s College, London, UK; Wellcome Foundation Building, King’s College Hospital, London, SE5 9PJ UK; Current address: Neurorehabilitation Center, RehaClinic, Bad Zurzach, Switzerland; Current address: University of Zurich, Zürich, Switzerland; Current address: Department of Neurology, Ghent University Hospital, Ghent, Belgium; Neurology and Headache Center, Munich, Germany

**Keywords:** Migraine, Trigeminovascular system, Nociception specific blink reflex (nBR), Pharmacology

## Abstract

**Background:**

The physiology and pharmacology of activation or perception of activation of pain-coding trigeminovascular afferents in humans is fundamental to understanding the biology of headache and developing new treatments.

**Methods:**

The blink reflex was elicited using a concentric electrode and recorded in four separate sessions, at baseline and two minutes after administration of ramped doses of diazepam (final dose 0.07 mg/kg), fentanyl (final dose 1.11 μg/kg), ketamine (final dose 0.084 mg/kg) and 0.9 % saline solution. The AUC (area under the curve, μV*ms) and the latency (ms) of the ipsi- and contralateral R2 component of the blink reflex were calculated by PC-based offline analysis. Immediately after each block of blink reflex recordings certain psychometric parameters were assessed.

**Results:**

There was an effect due to DRUG on the ipsilateral (*F*_3,60_ = 7.3, *P* < 0.001) AUC as well as on the contralateral (*F*_3,60_ = 6.02, *P* < 0.001) AUC across the study.

A significant decrement in comparison to placebo was observed only for diazepam, affecting the ipsilateral AUC. The scores of alertness, calmness, contentedness, reaction time and precision were not affected by the DRUG across the sessions.

**Conclusion:**

Previous studies suggest central, rather than peripheral changes in nociceptive trigeminal transmission in migraine. This study demonstrates a robust effect of benzodiazepine receptor modulation of the nociception specific blink reflex (nBR) without any μ-opiate or glutamate NMDA receptor component. The nociception specific blink reflex offers a reproducible, quantifiable method of assessment of trigeminal nociceptive system in humans that can be used to dissect pharmacology relevant to primary headache disorders.

## Background

The physiology and pharmacology of head pain underpins understanding of the various syndromes and development of new therapies for conditions such as migraine and cluster headache. Migraine is an episodic brain disorder that affects about 15–18 % of the population [1], is the most common cause of disability due to a neurological disorder [2] and has been estimated to be the most costly neurological disorder in the European Community at more than €27 billion per year [3]. Major therapeutic development, in the form of relatively specific and effective acute [4, 5] and preventive [6, 7] treatments of migraine have provided impetus to understand their mechanism of action. Since activation, or the perception of activation, of pain-coding trigeminovascular afferents underlies most disabling primary headaches [8], understanding human trigeminal mechanisms is essential for therapeutic development. The description of a method for activating the trigeminal system in humans to study pain-induced responses [9] has spurred many studies of this crucial pathway [10].

The blink reflex (BR) is a physiological, protective trigeminofacial reflex aimed at facilitating eyelid closure as a response to a threatening and potentially harmful stimulus [11]. In clinical practice the BR can be elicited when the skin innervated by the supraorbital nerve is electrically stimulated and a compound muscle action potential (CMAP) from the surface of the orbicularis oculi muscle is recorded. The afferent portion of BR is supplied by branches of the trigeminal nerve, and the efferent volleys are conducted by branches of the facial nerve. Suprasegmental influences on the BR are less well understood, but evidence from studies in patients with movement disorders, stroke and multiple sclerosis suggest input from the central cortex, basal ganglia and thalamus [12]. The reflex response consists of three components – R1, R2, and R3. R1 is mediated by exteroceptive A-beta fibers that project to low-threshold mechanoreceptive neurons in the pons [11, 13]. This response has an ipsilateral oligosynaptic reflex arc connecting the Gasserian ganglion to the pontine sensory nucleus of the trigeminal nerve and subsequently to the ipsilateral facial nucleus [14]. The R2 response is mediated by A-beta and A-delta fibers, projecting to wide dynamic range (WDR) neurons in the medulla including this of the spinal trigeminal nucleus [15]. R3 is mediated by A-beta and A-delta fibers and the precise connections are debated [16]. R3 and R2 are bilateral responses elicited by both noxious and innocuous stimuli. Only R2 can be elicited by selective activation of nociceptors [17]. Kaube and colleagues [9] developed a novel concentric electrode to allow specific study of nociceptive components of the R2 component of the reflex, the nociception-specific blink reflex (nBR).

It has been reported that fentanyl, an opioid receptor agonist, and diazepam both reduce the R2 component of the blink reflex [18]. Ketamine, despite its use in pain [19], has only infrequently been studied in migraine [9, 20], and most recently in migraine aura [21]. In this study we sought to explore the pharmacology of the nociceptive portion of the R2 response using the novel concentric electrode, and thus provide data in humans on some aspects of the pharmacology of this important synapse.

## Methods

### Demographics

The study was approved by The National Hospital for Neurology and Neurosurgery and The Institute of Neurology Joint Research Ethics Committee and Directorate for Research and Development. Male volunteers (*n* = 31) aged between 21 and 37 years underwent initial screening assessment, which included a careful history and physical examination, after signing an informed consent document that explained and described the study. Ten subjects were unsuitable because of intercurrent medical issues (*n* = 6) or for practical (*n* = 4) reasons, the latter being that they could not agree to attend on repeated occasions. Twenty-one non-smoking [22] subjects 27 ± 4 years old (mean ± SD) with unremarkable medical history, including no history of a headache disorder, normal physical examination and clinical laboratory tests, including negative urine drug screening, were recruited into the study. None of the volunteers received any prescribed medication for 30 days prior to the screening or after entering the study, for its entire duration. No medication was used during the 24 h prior to the recordings. Alcohol consumption was restricted to less than 10 units per week, with no intake for 24 h prior to and after each recording.

### Recordings

All recordings were carried out between 9.00 and 17.30, with at least one common to all investigator blinded to the treatment (JCAM), under similar conditions and using the same verbal instructions on each occasion. The subjects were lying supine, in a comfortable adjustable bed in a warm room with dim light, keeping their eyes closed during the recordings. When required, gentle pressure was applied on the eye lids by an eye pad, helping the subjects to relax, in order to avoid interference by eyelid fluttering and thus reduce noise. EMG recordings were obtained from bilateral gold-plated surface electrodes placed infraorbitally over the belly of the orbicularis oculi muscle (different) and the lateral orbital ridge (indifferent); acquisition band width 1 to 1000 Hz (in isolated cases the lower cut off frequency was increased from 1 to 20 Hz to avoid clipping of the signal due to pronounced EOG responses), sampling rate 2.5 kHz (750 data points/sweep), sweep length 300 ms, amplifier gain 10,000 X (micro 1401, Signal Version 2, Cambridge Electronic Design, UK).

### Stimulation

Blink reflexes were elicited with a custom built concentric electrode in using a printed circuit board design: central platinum cathode (D: 0.5 mm) and an external anode ring (D: 6 mm; thickness 1 mm), providing a stimulation area of 20 mm^2^, placed about 10 mm above the right supraorbital foramen [9]. Stimulation was performed with a constant current source (Stimulator DS7A Digitimer, UK) by delivering a train of three square wave monopolar (positive) pulses [23], each lasting 0.5 ms, with an interpulse interval of 4 ms, maximum voltage 400 V. The current intensity for nociception-specific stimulation was 0.5–1.8 mA (mean 0.95 ± 0.19 mA), representing 2–4 times individual threshold for pin-prick perception (mean 0.31 ± 0.06 mA) [24]. Each session (of a total of four) consisted of four blocks (one at the base line and one after each dose, see below), separated by 10 min. Each block consisted of seven sweeps with an inter-stimulus interval of 12–18 s (randomized, uniform distribution). Grounding to earth was secured by a moist wrist band electrode or by a standard disposable ECG gel electrode placed over the mastoid protuberance.

### Study design

In a single-center, randomized, double-blinded, placebo-controlled, crossover, four-arm study, the sensitivity of the blink reflex, as a marker of the central activity of diazepam, fentanyl and ketamine, was assessed versus specific CNS related parameters.

The volunteers were randomly assigned to receive a sequence of four treatments each, on four different occasions, separated by a washout period of a minimum four days.

### Treatments

The subjects were cannulated and received either a ramped dose of intravenous diazepam: initial dose 0.03 mg/kg with two subsequent increments of 0.02 mg/kg each [25]; intravenous fentanyl: initial dose 0.37 μg/kg with two additional increments of 0.37 μg/kg each [26, 27]; intravenous ketamine: initial dose 0.028 mg/kg with two subsequent identical increments of 0.028 mg/kg [28–30], or intravenous placebo. Placebo was given as pure saline infusion and the active drugs were mixed with saline solution, all administrated from a 50 ml volume syringe. Each infusion (dose increment), including placebo increments, was delivered over two minutes (300 ml/h) using an IVAC (TIVA ALARIS Medical Systems, Hampshire, UK) infusion pump.

### Data collection

Continuous telemetric ECG, pulse, oxygen saturation and respiratory rate monitoring and intermittent (every 10 min) blood pressure measurements were performed during and two hours following the experiments (Philips C3 Patient Monitor 862474, USA). During every session, assessments of the blink reflex (see *Statistical analysis*) took place at the base line and two minutes after each dose increment, respectively.

#### Psychometric tests

Immediately after each block of blink reflex recordings certain psychometric parameters were objectively assessed by measuring the speed of reaction and the accuracy of responses to visual stimuli (as described below) and subjectively, using a computer adapted (Matlab 5.3, The MathWorks, Nattick, MA, US) version of Bond Lader Rating Scale [31]. The test consisted of 16 pairs of opposite adjectives, each pair presented separately, with the opposite words delineating a 155 mm long and 20 mm wide bar, horizontally centred on a 15 in. Dell computer screen, positioned at a comfortable distance and in an appropriate visual angle. The subjects were instructed to mark the extent to which they felt the two opposite adjectives corresponded to their state of mind (i.e. closer to one quality and more distanced from its opposite) using a cross-hair cursor controlled by a mouse. The distance in mm was measured, the scores reversed and then analyses were made on three different fields, measuring alertness, calmness and contentedness.

To monitor objectively the subjects’ level of alertness a special function was edited in Matlab using Cogent 2000 (version 1.24) to present fifty pairs of either identical or almost identical (four identical digits, one different) five digit figures (Arial size 100), on the same computer screen described above. Each figure was presented for 200 ms with an inter-figure interval of 400 ms. The subjects were instructed to hit a key (arbitrarily chosen on a keyboard) when the paired figures were identical and a different key when the paired figures were not identical, as soon as possible, after the second figure in each pair was presented. The same keys being used for identical respective different events through the entire test. The reaction time was monitored up to 1000 ms after the presentation of the second figure in each pair. Slower responses were computerized as errors and excluded from the averaged reaction time in a final (after each test) score of error rate and mean reaction time. The subjects were allowed to familiarize with both tests (reaction performance and Bond Lader Rating Scale) before the first experiment.

### Data analysis

The first recording sweep on all recordings was discarded to avoid contamination by initial startle responses. The onset latency (ms) and the area under the curve AUC (μV x ms) of the R2 component (rectified EMG) between 35 and 95 ms were calculated by PC-based offline analysis with custom written software programmed in Matlab. Data were recorded on an Excel spreadsheet and checked before the database was locked and the randomisation code was broken. Summary data are presented as mean ± SEM. Reproducibility between visits was tested with Cronbach’s alpha. Data were analysed initially using an ANOVA with repeated measures for an effect of *TIME* and *DRUG* treating each study episode as independent. To examine the three independent hypotheses regarding opioid, benzodiazepine and glutamatergic involvement in the nociception-specific blink reflex the ANOVA with repeated measures regarded treatment episodes as related within subjects and examined the DRUG main effect with Bonferroni correction (SPSS for Windows). If Mauchly’s test of sphericity was violated, we made appropriate corrections to degrees of freedom according to Greenhouse–Geisser. Significance was assessed at the *P* < 0.05 level.

## Results

All subjects completed the entire cross-over with no drop-outs. The volunteers had a mass of 72 ± 8 kg and were 1.8 ± 0.1 m in height.

### Baseline data

Baseline blood pressure (mean arterial pressure, MAP = 2/3* Diastolic + 1/3 *Systolic) was 87 ± 2 mmHg, heart rate 62 ± 2 beats/min, respiratory rate was 20 ± 1 breaths per minute and oxygen saturation 98 ± 1 %.

#### Blink reflex

**Fig. 1:**
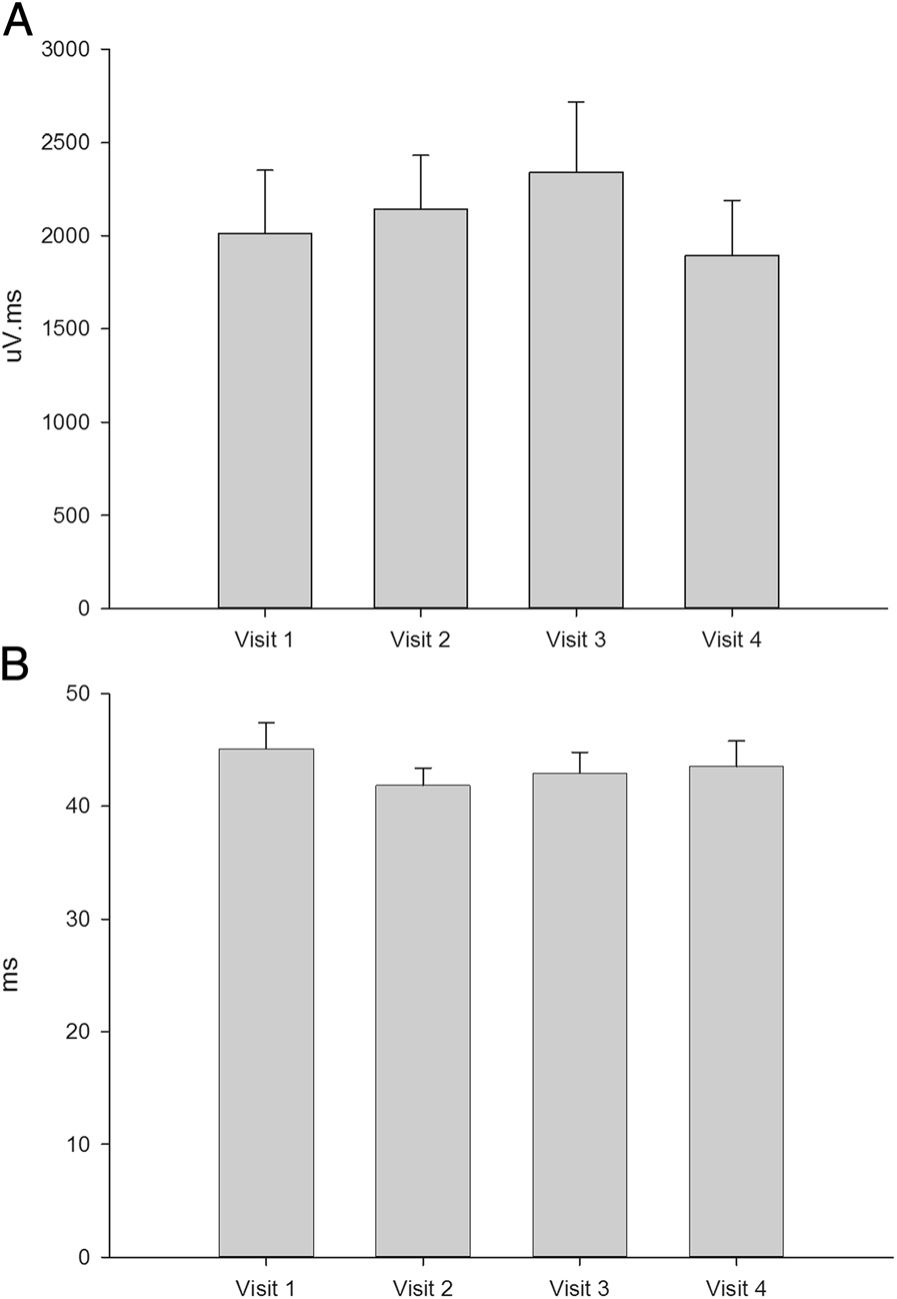
Baseline measures of the nociception specific blink reflex from twenty-one volunteers on four separate visits showing a high degree of reproducibility of intra-patient measures for the area-under-the-curve (AUC, μV.ms, Panel **a**) and latency (ms, **b**) for the ipsilateral responses. Contralateral responses were similarly reproducible (see Results)

The baseline ipsilateral AUCs (μV.ms) for the four visits (ketamine, diazepam, fentanyl and placebo) were (mean ± SEM): 2082 ± 283, 1892 ± 299, 2340 ± 376 and 2011 ± 341, respectively. These were highly reproducible (alpha = 0.85, Fig. 1a). The baseline ipsilateral latencies (ms) for the four visits were: 41.8 ± 1.5, 43.5 ± 2.3, 42.9 ± 1.9 and 45.1 ± 2.3, respectively. Again these were highly reproducible (alpha = 0.86, Fig. 1b). The baseline contralateral AUCs (μV.ms) for the four visits were: 1364 ± 239, 1156 ± 188, 1461 ± 324 and 1336 ± 306, respectively. The baseline contralateral latencies (ms) for the four visits were 47 ± 2, 47 ± 3, 46 ± 3 and 46 ± 3, respectively.

**Fig. 2:**
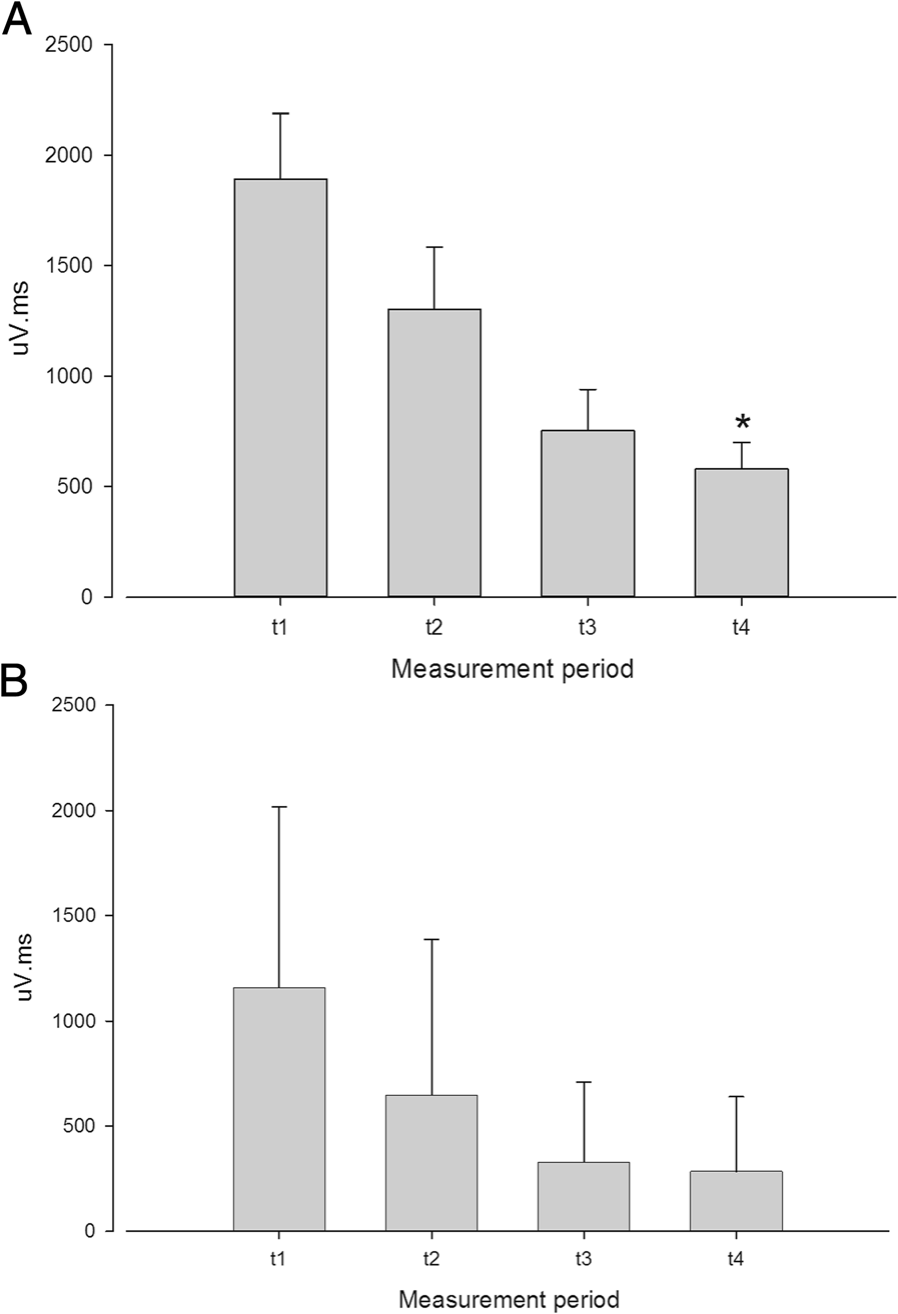
The effect of the benzodiazepine receptor agonist diazepam on the nociception specific blink reflex, showing a significant (*, *P* < 0.05) reduction in the AUC (μV.ms) ipsilateral to stimulation (Panel **a**), and no significant effect contralaterally (**b**). Measurement periods t1 through t4 reflect the data collection epochs

#### Neuropsychological tests

From the Bond-Lader scale the alertness (ketamine 30 ± 4, diazepam 23 ± 3, fentanyl 25 ± 3 and placebo 25 ± 4), calmness (28 ± 4, 24 ± 4, 27 ± 4 and 23 ± 3) and contentedness (25 ± 3, 21 ± 3, 21 ± 3 and 22 ± 3) scores were calculated. Reactions times (ms) for the four visits were: 479 ± 15, 447 ± 14, 406 ± 17 and 379 ± 18, respectively. Error rates for the four visits were: 13 ± 2, 15 ± 2, 17 ± 2 and 16 ± 2, respectively.

### Overall analysis

#### Electrophysiology

The ipsilateral AUC demonstrated a reduction with time during the four measurement points in the overall analysis (*F*_2.0,162.4_ = 15.6, *P* < 0.001). There was a significant reduction in ipsilateral AUC attributable to the variable *DRUG* (*F*_3,80_ = 3.1, *P* = 0.03). For the contralateral AUC there was a reduction with time (*F*_2.3,175.1_ = 9.7, *P* < 0.001) without a significant *DRUG* effect for the overall analysis (*F*3,80 = 2.6, *P* = 0.057). There was no significant effect on the ipsilateral (*F*_3,62_ = 2.4, *P* = 0.064) or contralateral (*F*_3,47_ = 1.1, *P* = 0.36) latency attributable to *DRUG*. Latency data were not further analysed.

#### Neuropsychology

From the Bond-Lader scale for the alertness score (*F*_3,80_ = 1.02, *P* = 0.39), the calmness score (*F*_3,80_ = 0.79, *P* = 0.51), or the contentedness score (*F*3,80 = 0.36, *P* = 0.78), there was no effect of *DRUG*, although there was a subjective reduction in alertness over the measurement points (*F*_1.8,141.3_ = 48.1, *P* < 0.001) not seen with the calmness or contentedness scores. The reaction time was not affected across the measurement points (*F*_3,240_ = 0.19, *P* = 0.90) or by *DRUG* (*F*_3,80_ = 2.1, *P* = 0.10). The error rates were constant across time (*F*_3,237_ = 1.3 *P* = 0.28) and not altered by *DRUG* (*F*_3,79_ = 0.59, *P* = 0.62).

### Treatment effects

There was a treatment effect across the study due to *DRUG* (*F*_3,60_ = 7.3, *P* < 0.001) in the ipsilateral AUC, that was further explored. Similarly there was a significant overall effect of *DRUG* on contralateral AUC (*F*_3,60_ = 6.02, *P* < 0.001).

#### Placebo

The ipsilateral AUC was constant for the placebo infusion at 2011 ± 341, 1907 ± 290, 2056 ± 333 and 1731 ± 258 (μV.ms), respectively, with alpha = 0.96. Similarly the contralateral AUC was constant at 1336 ± 306, 1305 ± 288, 1363 ± 309 and 1187 ± 307, respectively, and again with alpha = 0.96.

#### Ketamine

The AUCs were 2144 ± 288, 2445 ± 335, 2480 ± 328 and 1955 ± 256 (μV.ms), respectively. Ketamine had no effect on the ipsilateral AUC when compared with placebo (*P* = 0.84). Contralateral AUCs were 1377 ± 245, 1662 ± 304, 1630 ± 289 and 1410 ± 263, respectively. There was no significant effect of ketamine on contralateral AUC (*P* = 1.0).

#### Fentanyl

For fentanyl ipsilateral AUCs were 2340 ± 376, 2219 ± 294, 1852 ± 357 and 1673 ± 334 (μV.ms), respectively. There was no effect of fentanyl on ipsilateral AUC compared to placebo (*P* = 1.0). Contralateral AUCs were 1461 ± 324, 1515 ± 359, 1158 ± 295 and 849 ± 218, respectively. There was no significant effect of fentanyl on contralateral AUC (*P* = 1.0).

#### Diazepam

For diazepam ipsilateral AUCs were 1892 ± 299, 1301 ± 284, 756 ± 185 and 580 ± 120 (μV.ms), respectively. There was a significant decrement in ipsilateral AUC with diazepam compared to placebo (*P* = 0.047, Fig. 2). Contralateral AUCs were 1156 ± 188, 647 ± 161, 328 ± 83 and 284 ± 78. The effect of diazepam did not reach statistical significance for the contralateral AUC (*P* = 0.07).

## Discussion

The study demonstrates an effect of benzodiazepine receptor modulation on the nociception-specific blink reflex without a detectable μ-opioid receptor or ketamine (glutamate-NMDA receptor) component in this model. The baseline effects were highly reproducible from session to session within subjects for four visits, consistent and extending previous observations [23], demonstrating the robustness of the method and thus its potential for serial assessments after suitable intervention.

A study by Kaube and colleagues using the novel concentric surface electrode [9] demonstrated an increase of the R2 component greater than 500 % on the headache side during acute migraine attacks [32]. An interictal deficiency in habituation of the nBR in migraine patients and also in asymptomatic individuals with a family history of migraine [33] emphasizes the potential for this reflex in understanding migraine pathophysiology.

### Nociception specific blink (nBR) reflex

The nBRr [9] R2 component is increased by more than 500 % in acute migraine [32]. This response seemed specific to migraine and was not observed in patients with acute frontal sinusitis [34], or in hypnic headache [35]. Interestingly, the nBR does not behave differently from controls in cluster headache in terms of habituation [36], although there is certainly lateralized facilitation [37]. The observation of an interictal persistent change raises the possibility of an endophenotypic marker for migraine [33]. This is important when one considers migraine as a substantially centrally mediated genetic disorder [8]. To this end triptans, serotonin 5-HT_1B/1D_ receptor agonists [38], which are highly effective in migraine [39], have also been reported to affect the nBR. Aspirin and zolmitriptan suppressed the reflex by 68 and 78 %, respectively, in migraineurs during an acute attack [40]. Moreover, the A_1_ receptor agonist GR79236 [41], which is effective in animal models of trigeminovascular nociception [42] and in acute migraine [43], also significantly inhibited the nBR. Taken together the data suggest that the model is helpful in understanding likely effects of various agents in migraine.

### nBR human pharmacology

It has been reported that fentanyl, an opioid receptor agonist, and diazepam, which acts by potentiating the GABA mediated inhibitory effect, both reduce the R2 component of the blink reflex [18]. Dauthier and colleagues [44] found that fentanyl 3 μg/kg did not alter the R2 response of the blink reflex. The dose used was slightly higher than the one employed here. Interestingly, fentanyl at a dose of 100 μg intramuscularly also failed to affect the R2 component of the blink reflex [45]. Taken with our studies the data suggest that opioid mechanisms are less important in the trigeminal system, although the latter studies were conducted with traditional electrodes with their limitations. In contrast, with the more conventional electrodes, it has been shown that zolmitriptan reverses blink reflex changes seen in migraine [46]. It is interesting that ketamine, despite its use in pain [19] did not alter the nBR. NMDA receptors, as a therapeutic target for migraine therapy have been discussed by Nicolodi and Sicutieri [20] who found that ketamine was effective as acute and preventive migraine treatment, when compared to placebo. Interestingly, when used in migraine with hemiplegic or prolonged aura, ketamine is useful for the aura component but not for the pain [21, 47].

### Trigeminocervical nociceptive pharmacology

The new data are broadly consistent with those from lower species with some caveats [38]. Diazepam modulates the GABA_A_ receptor, and it has been clearly shown in animals that GABA_A_ mechanisms can modulate nociceptive trigeminovascular transmission at the second order synapse in the trigeminocervical complex [48]. Similarly, more direct evidence can be seen with studies of the benzodiazepine receptor in the anaesthetized cat with midazolam inhibiting transmission at a site where the effect is reversed by flumazenil [49]. Interestingly, in humans, the changes induced by diazepam on the R2 component of the blink reflex, were only partially reversed by flumazenil [18]. There is also a body of evidence from studies of neurogenically-mediated dural mechanisms for a role of GABA receptors in this system [50–52]. In contrast to the new data, animal studies do suggest the existence of the mu-opioid receptor in the trigeminocervical complex [53]. Similarly, there is certainly glutamatergic transmission at the trigeminocervical complex [54–56] and again effects in dural models [57, 58], suggesting a dose or more crucial difference in terms of human trigeminal nociceptive transmission.

It might be argued that diazepam’s influence on the R2 component of the blink reflex is due to a muscle relaxant effect. However, this was not observed in the current study with fentanyl. Moreover, previous work demonstrated a significant reduction of the R2 response with diazepam doses that did not alter the R1 response [44, 59]. Since R1 and R2 are mediated by the same muscle groups, a muscle relaxant effect as an explanation for the R2 response reduction seen after diazepam administration is not likely.

### Limitations

The study has some important limitations that need consideration. When studying human volunteers it is imperative to consider safety. Such considerations limit the use of sedative treatments, particularly opioids whose major significant short term side effect is respiratory depression. Ketamine may have been under-dosed, although the results are consistent with other published data [47]. Krystal and colleagues [30] found that doses as low as 0.1 mg/kg caused psychiatric side effects in healthy volunteers when assessed by the Brief Psychiatric Rating Scale. Therefore, we chose a slightly lower dose (0.084 mg/kg), aiming, for both ethical and scientific purposes, to determine doses with minimal side effects, which is important since there are a paucity of human dose ranging studies of ketamine in this use. Ketamine is a short acting drug [29] with an effect we would expect to last no more than minutes and a small therapeutic window [26]. These pharmacokinetics favor a cumulative dose regimen as employed here [26], with test batteries performed immediately after or within 10 min after each dose increase. Our findings are also consistent with data showing that ketamine at a slightly lower dose than the total used here was effective as reducing the anxiety without altering the pain scores [28]. It is important to note the outcome of the study is limited to the pharmacology of the response elicited by the nBR, and cannot be generalized to the entire trigeminal system. Moreover, by using the nBR rather than a pain scale, we are limited in drawing inferences about how subjects may rate pain effects with these drugs.

## Conclusions

We have shown in a carefully conducted three-way crossover study in volunteers a reduction of the nociception specific blink reflex with the benzodiazepine receptor agonist diazepam. We have seen no clear effect of fentanyl, the mu-opioid receptor agonist or ketamine, the non-competitive glutamate NMDA-receptor blocker. The study showed a highly reproducible baseline from week to week within subjects. The nociception specific blink reflex offers a reproducible, quantifiable window with which to examine the trigeminal nociceptive system in humans. Studies of preventive medicines and putative new anti-migraine treatments may provide useful insights into both the pathophysiology of migraine and help accelerate medicine development.
